# Influence of Voxel Size on CBCT Images for Dental Implants Planning

**DOI:** 10.1055/s-0041-1736388

**Published:** 2021-12-13

**Authors:** Ricardo Kehrwald, Hebert Sampaio de Castro, Samira Salmeron, Ricardo Alves Matheus, Gustavo Machado Santaella, Polyane Mazucatto Queiroz

**Affiliations:** 1 Department of Dentistry, Area of Implantology, Ingá Center University Maringa, Parana, Brazil; 2 Department of Oral Medicine, Division of Oral Radiology, State University of Londrina, Londrina, Parana, Brazil; 3 Department of Diagnosis and Oral Health, Division of Oral Radiology, University of Louisville, Louisville, Kentucky, United States; 4 Department of Dentistry, Division Oral Radiology, Area of Oral Radiology, Ingá Center University Maringa, Parana, Brazil

**Keywords:** cone beam computed tomography, dental implants, diagnosis, dimensional measurement accuracy, implant planning, jaw edentulous, mandible

## Abstract

**Objective** This study was developed to evaluate the influence of voxel size on bone measurements for implant planning.

**Materials and Methods** The research was performed by using edentulous synthetic human mandibles with different levels of bone resorption. For each mandible, height and bone thickness were measured with a digital caliper. The PaX-i3d device was used to acquire the volumes of the five mandibles, with 50kVp, 4 mA, and a voxel size of 0.08 mm. After the acquisition, the images were reconstructed in the software CS three-dimensional Imaging, with four different sizes of voxels: 0.1, 0.2, 0.3, and 0.4 mm. All volumes were analyzed by a single evaluator who performed measurements to obtain bone height and thickness, using the reference points that were considered in obtaining the gold standard. The data were analyzed by ANOVA with a significance level of 5%.

**Results** There was no significant difference in the measurements obtained with different voxel sizes, both for bone height measurements and bone thickness. There was no statistically significant difference in measurements in thickness in comparison to the gold standard.

**Conclusion** When necessary, to measure height and bone thickness, it is possible to recommend voxel images of larger size (0.40 mm) without compromising the quality of the patient's clinical planning.

## Introduction

In any area of dentistry, the correct planning of the case is fundamental for the satisfactory result, and in implantology, it is not different. Imaging exams are complementary in the diagnostic process, and they are an essential resource for treatment planning. An imaging technique that allows the evaluation of bone quality, height, and thickness is sought as the ideal, in addition to enabling the analysis of the relationship of implant sites with vital anatomical structures. 1 Complementary exams are essential to avoid transoperative and postoperative complications, such as hemorrhage due to artery injury, paresthesia due to nerve tissue damage, poor positioning of the implant, and compromising its stability among others. 2

However, all two-dimensional exams have limitations inherent to the technique that can cause image misrepresentation resulting in inaccuracies of information and measurements for treatment planning. 3 In contrast, cone beam computed tomography (CBCT) refers to a diagnostic imaging resource that is capable of obtaining data and reconstructing them volumetrically, enabling the analysis of structures in different visualization planes, with real size dimensions and without image overlap. 4

The CBCT device is compact and the patient is positioned seated or standing for the examination. A tube-detector system performs a rotation around the patient's head, and at each determined degree of rotation, the device acquires a base image of the patient. After the end of the rotation, this sequence of base images is reconstructed to generate the three-dimensional volume through a specific software installed on a computer coupled to the CBCT unit. 5 6

In the evaluation of hard tissues, CBCT is superior to conventional CT scans due to the size of the voxel. The voxel is a volume element; it is the smallest unit of a tomographic image. The three-dimensional images are composed of voxels, which have the size determined by their height, width, and depth. The isotropic nature (of the same size in all its dimensions) of voxels in CBCT images provides the same quality as the original image in the multiplanar reconstructions. 7

In this context, the size of the voxel is an important factor in the spatial resolution of the image. The smaller the voxel size, the higher the spatial resolution; however, the larger the file size and the longer the time it takes to reconstruct the image. Due to the importance of the size of the voxel in the spatial resolution of CBCT volumes, the present study was developed to evaluate the influence of the size of the voxel in the bone measurements used for dental implants planning.

## Materials and Methods

### Sample and Gold Standard Group

**Fig. 1:**
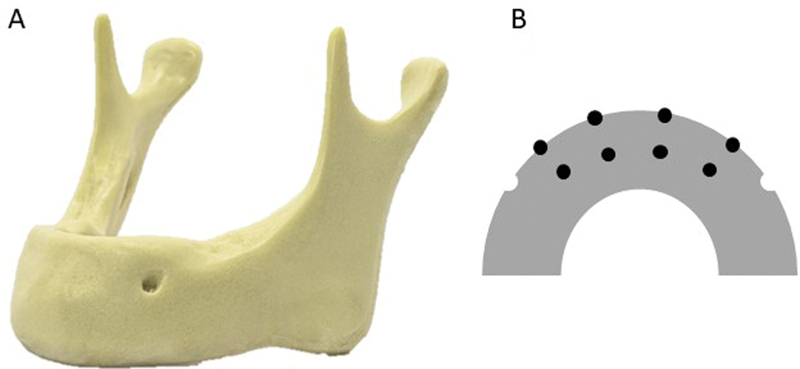
( **A** ) Synthetic polyurethane mandible used for image acquisition. ( **B** ) A representative scheme of an occlusal view of a mandible, interforaminal region, indicating the marking of the reference points.

The research was performed by using five synthetic human mandibles (Nacional Ossos, Jaú, Brazil). The mandibles are made of high-density rigid polyurethane ( Fig. 1A ). Models of edentulous mandibles with four levels of bone resorption were used.

As reference points, eight markings were made with a permanent marker pen. In the anterior region of the mandible, between both mental foramen, four equidistant markings were made in the superior cortical of the alveolar process (occlusal) and four markings in the buccal cortical ( Fig. 1B ). Then, with the use of a spherical carbide bur no. 1/2 (KG Sorensen, Cotia, Brazil), in low rotation, drilling was made at the marked points, inserting the entire active tip of the bur, using a straight handpiece (Kavo Kerr, Joinville, Brazil).

For each mandible, bone height and thickness were measured with a previously calibrated digital caliper (Starrett no. 727–6/150, Massachusetts, United States). The perforations made were used as the references. For bone height, we considered the marking in the upper cortical of the alveolar process aligned to the lower border of the mandible, and for the bone thickness, it was considered the marking of the buccal cortical to the lingual cortical.

### Acquiring the Volumes

The PaX-i3d unit (Vatech, Hwaseong, South Korea) was used to acquire the CBCT volumes of the five mandibles. The images were acquired with energy parameters of 50 kVp and 4 mA, as determined in pilot study, field of view of 50 × 50 mm, and a voxel size of 0.08 mm.

**Fig. 2:**
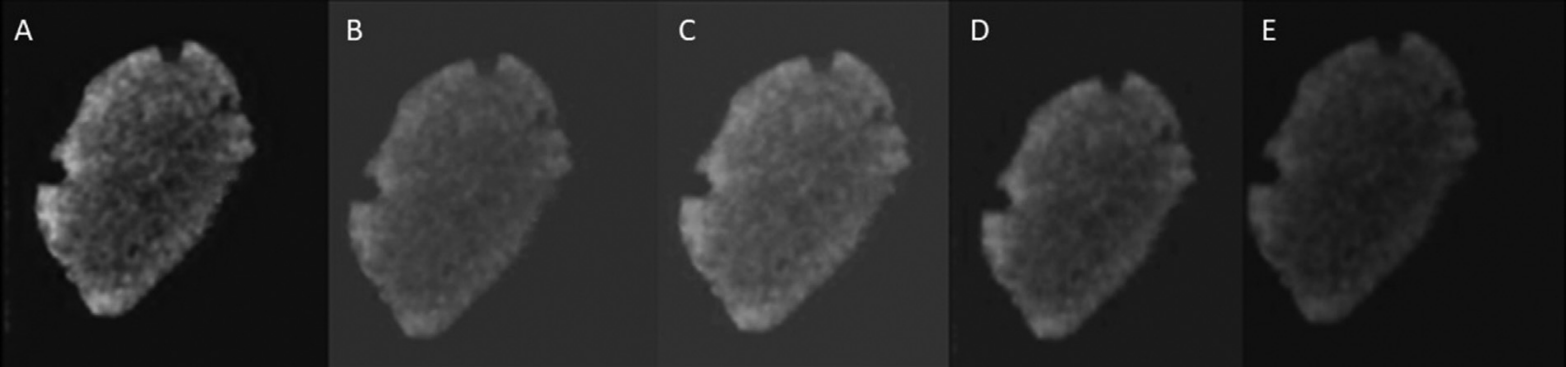
Cross-sections with different voxel sizes ( **A** ) 0.08, ( **B** ) 0.1, ( **C** ) 0.2, ( **D** ) 0.3, and ( **E** ) 0.4 mm.

After the acquisition, the images were reconstructed in the CS 3D Imaging software (Carestream Dental LLC - Atlanta; Georgia, United States), obtaining new volumes with four different voxel sizes: 0.1, 0.2, 0.3, and 0.4 mm ( Fig. 2 ).

### Evaluation of Images

**Fig. 3:**
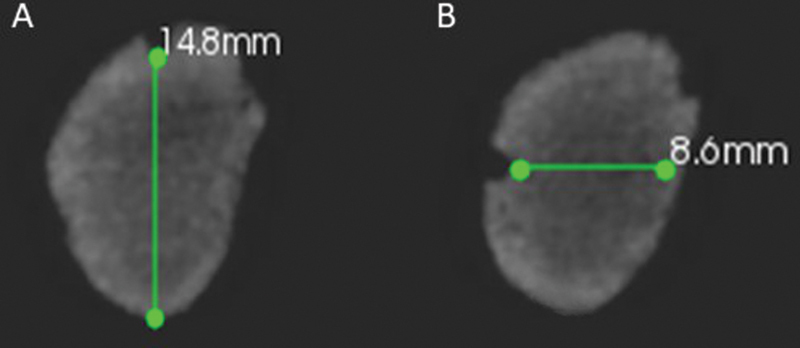
Cross-sectional images with the markings of ( **A** ) height and ( **B** ) bone thickness.

All volumes were analyzed by a single experienced evaluator, previously calibrated, who performed the measurements to obtain bone height and thickness ( Fig. 3 ), using the reference points that were considered in obtaining the gold standard. The height was demarcated at the lower border of the concavity of the reference point of the superior cortical perpendicular to the base of the mandible. The bone width was marked at the posterior edge of the concavity of the reference point in the vestibular cortical to the lingual cortical, parallel to the horizontal plane. The images were evaluated dynamically in the CS 3D software so that the evaluator could make use of the features of brightness, contrast, and zoom as needed. Thirty days after the initial evaluation, 30% of the sample was reevaluated to confirm the reproducibility of the analysis.

### Statistical Analysis

The measurements of height and bone thickness obtained in the CBCT images were tabulated. The data were submitted to statistical analysis in which a one-way Analysis of Variance (ANOVA) was performed with a significance level of 5%, to compare the measurements of the images obtained with different voxels and with the gold standard. For reproducibility analysis, the Intraclass Correlation Index (ICC) was calculated. The analyses were made in BioEstat software (Mamirauá Foundation, Belém, Brazil) and MedCalc (MedCalc Software, Oostende, Belgium).

## Results

The data obtained are presented in Table 1 . The ICC value for reproducibility was 0.9968.

**Table 1:** Mean values of measurements in millimeters (mm) of height and thickness bone of gold standard and of images with different voxel sizes (mm) to different mandibles (M1, M2, M3, M4, and M5)

		GS	0.08	0.10	0.2	0.3	0.4
M1	Height	26.83	24.48	24.33	24.43	24.45	24.18
	Thickness	9.85	8.77	8.63	8.18	8.78	8.73
M2	Height	19.40	19.77	19.00	18.95	19.33	19.38
	Thickness	10.70	10.58	10.48	10.83	10.55	10.88
M3	Height	15.30	15.18	15.13	15.18	15.18	15.25
	Thickness	14.30	12.80	12.73	13.03	12.93	13.05
M4	Height	10.33	9.88	9.75	10.00	10.05	9.98
	Thickness	11.85	11.25	11.28	11.05	11.25	11.28
M5	Height	10.30	9.43	9.33	9.73	10.08	9.30
	Thickness	9.40	8.90	9.18	8.73	8.60	8.73

Abbreviation: GS, gold standard.

When compared the measurements of the bone height obtained in the images with different voxel sizes, no statistically significant difference was observed for the measurements of the images obtained with the different voxel sizes ( *p* = 0.9991). The measurements also showed no statistically significant difference compared with the gold standard ( *p* = 0.9959).

No statistically significant difference was observed between measurements of bone thickness performed on images obtained with different voxel sizes ( *p* = 0.9986), and there was no significant difference in these measurements compared with the gold standard ( *p* = 0.9447).

Both for height and bone thickness, although there is no statistically significant difference, usually, CBCT underestimated the measurements.

## Discussion

The correct diagnosis and planning of the case are directly associated with the success rates for the treatment of the patient. The practitioner can use some methods for planning dental implants, such as panoramic radiography or CBCT scans. CBCT images provide three-dimensional information about the implant site and adjacent anatomical structures, and they allow viewing of the area of interest in precise sections or slices. 8

To obtain the CBCT volumes with quality, some parameters should be selected before the examination such as the voxel size. The voxel is the smallest unit of a volumetric image, and it has a fundamental importance in the image, as it is related to its spatial resolution. In theory, the smaller the voxel size is the sharper the image tends to be. 9 However, the voxel size may also influence the amount of image noise, even in reconstructed volumes, 10 as in this study, which can have repercussions on image quality. In addition, other factors also influence image quality, such as contrast resolution, rotation time, and reconstruction technique, 11 for example.

For diagnostic purposes, studies show that there is an influence of the voxel size on the ability to detect conditions such as external root resorption, 12 root fractures, 12 13 14 measurement of dental volume, 15 and sharpness in the visibility of anatomical structures, 16 for example. In general, the authors report that images obtained with smaller voxel sizes are more accurate for the diagnosis of these conditions. In contrast, Kobayashi-Velasco et al 17 and Sönmez et al 18 who evaluated the influence of the voxel size on the diagnosis of root and alveolar fracture and diagnosis of external root resorption, respectively, did not observe the influence of voxel size in their studies. Considering the possible presence or absence of voxel size influence on diagnostic tasks, the present study was developed to evaluate whether voxel size interferes with the accuracy of linear measurements on CBCT images.

The lack of influence on the diagnosis may be associated with the acquisition of images that present enough high spatial resolution, as it happened in the studies by Kobayashi-Velasco et al 17 and Sönmez et al, 18 in which the authors did not observe the influence of voxel size in the detection of conditions. However, the largest voxel used in these studies was 0.20 mm. Also, in the study by Yilmaz et al, 19 the authors did not find any influence of voxel size (range = 0.10 to 0.20 mm) in the measurement of residual volume of filling material in root canals. In the study by Dong et al, 20 the authors evaluated the influence of voxel size on the detection of alveolar bone defects. Among the images performed with voxel of 0.125 and 0.20 mm, no significant differences were observed. However, both protocols presented differences compared with the images obtained with 0.40 mm.

In the present study, regardless of the voxel size used to reconstruct the image, no significant difference was observed among the protocols, with voxel variation from 0.08 to 0.4 mm. The same was observed in the study by Costa et al, 21 in which the different voxel sizes (range = 0.125–0.40 mm) did not influence the accuracy of the measurement of the dimensions of the mandibular condyle, and in the study by Waltrick et al., 7 in which the authors observed that the voxel sizes studied (0.20, 0.30, and 0.40 mm) did not influence the linear measurement in the molar region and the identification of the mandibular canal in human jaws. Evidencing the possibility of using CBCT images in planning for dental implants, as presented in the systematic review by Fokas et al. 22

Thus, it is essential to consider that the presence or not of influence of voxel size in the diagnosis is associated with the diagnostic task in question. This is because the image quality may or may not have impacted the diagnosis. It is important to consider the relationship between the voxel size of the image and the size of the structure being evaluated. Since, when this area is smaller than the voxel size, there will be a representation only of the averaged values of adjacent structures, losing the faithful representation of the limits of the evaluated structure, as pointed out in the study by Melo et al. 23 In bone measurement tasks, there are no limits as precise or difficult to detect as it is in the detection of a root fracture line, or linear measurement of external root resorption, 18 for example.

Another consideration is the amount of noise in the CBCT image. When an image is obtained with a smaller voxel size, there will be a lower capacity of that voxel in detecting X-ray photons, which will result in more significant image noise. Thus, the images with smaller voxel present higher spatial resolution, however, noisier images. 24 Queiroz et al 12 reconstructed images with different voxel sizes and observed a greater amount of noise in the reconstructed images compared with the image originally obtained. Noise can compromise image quality. Thus, in diagnostic tasks that do not necessarily require high resolution, such as in the measurement of relatively large dimensions, there may be compensation in the resolution and noise parameters, causing no significant interference of the spatial resolution of the image, so that, regardless of the size of the voxel, it will be possible to perform accurate measurements.

It is not only by the influence of voxel size on the spatial resolution of the image and on the amount of image noise that the practitioner must be aware of this parameter, considering his diagnostic task. The voxel size also has an impact on the reconstruction time of the image, so the smaller voxel requires longer working time. And, in some devices, a change in voxel size may result in changes in exposure factors, resulting in greater exposure of the patient to obtain higher resolution images. 23 Or, voxel size may be associated with the size of the field of view, which may also influence the radiation dose and the amount of image noise. 25 In the present study, because it is an in vitro study, there are limitations such as the use of synthetic jaws that does not exactly reproduce the clinical reality. On the other hand, precisely because it is an in vitro study, it is possible to obtain images with the same exposure parameters and the same FOV size, eliminating possible influences of these variables on the studied factor.

Considering the need for measurement in Implantology, voxel size should not be a significant parameter to decide before the CBCT imaging. Thus, when necessary, it is possible to recommend voxel images of larger size (0.40 mm) that will imply shorter reconstruction time and smaller file size, and in some cases, even a lower radiation dose, without compromising the quality of the patient's clinical planning.
